# The effectiveness of complementary manual therapies for pregnancy-related back and pelvic pain: A systematic review with meta-analysis

**DOI:** 10.1097/MD.0000000000004723

**Published:** 2016-09-23

**Authors:** Helen Hall, Holger Cramer, Tobias Sundberg, Lesley Ward, Jon Adams, Craig Moore, David Sibbritt, Romy Lauche

**Affiliations:** a Australian Research Centre in Complementary and Integrative Medicine (ARCCIM), University of Technology Sydney, Sydney; b Faculty of Medicine, Nursing and Health Sciences, School of Nursing & Midwifery; Monash University, Frankston, Australia; c Department of Internal and Integrative Medicine, Kliniken Essen-Mitte, Faculty of Medicine, University of Duisburg-Essen, Essen, Germany; d Department of Neurobiolgy, Care Sciences and Society, Karolinska Institutet; e The Integrative Care Science Centre, Stockholm, Sweden; f Nuffield Department of Orthopaedics, Rheumatology and Musculoskeletal Sciences, University of Oxford, UK.

**Keywords:** back pain, complementary and alternative medicine, manual therapy, pelvic pain, pregnancy

## Abstract

**Background:**

Low back pain and pelvic girth pain are common in pregnancy and women commonly utilize complementary manual therapies such as massage, spinal manipulation, chiropractic, and osteopathy to manage their symptoms.

**Objective:**

The aim of this systematically review was to critically appraise and synthesize the best available evidence regarding the effectiveness of manual therapies for managing pregnancy-related low back and pelvic pain.

**Methods:**

Seven databases were searched from their inception until April 2015 for randomized controlled trials. Studies investigating the effectiveness of massage and chiropractic and osteopathic therapies were included. The study population was pregnant women of any age and at any time during the antenatal period. Study selection, data extraction, and assessment of risk of bias were conducted by 2 reviewers independently, using the Cochrane tool. Separate meta-analyses were conducted to compare manual therapies to different control interventions.

**Results:**

Out of 348 nonduplicate records, 11 articles reporting on 10 studies on a total of 1198 pregnant women were included in this meta-analysis. The therapeutic interventions predominantly involved massage and osteopathic manipulative therapy. Meta-analyses found positive effects for manual therapy on pain intensity when compared to usual care and relaxation but not when compared to sham interventions. Acceptability did not differ between manual therapy and usual care or sham interventions.

**Conclusions:**

There is currently limited evidence to support the use of complementary manual therapies as an option for managing low back and pelvic pain during pregnancy. Considering the lack of effect compared to sham interventions, further high-quality research is needed to determine causal effects, the influence of the therapist on the perceived effectiveness of treatments, and adequate dose–response of complementary manual therapies on low back and pelvic pain outcomes during pregnancy.

## 1 Introduction

Low back pain (LBP) and pelvic girth pain (PGP) are common in pregnancy and can have a significant impact on the woman's quality of life.^[1–3]^ LBP is characterized by pain between the 12th rib and the gluteal fold, whereas PGP is typically experienced in the vicinity of the sacroiliac joints.^[4]^ The underlying etiology of pregnancy-related back and pelvic pain is not fully understood. Current theories suggest that the symptoms may be related to changes in posture during pregnancy (increased lumbar lordosis), and increases in weight and instability of the pelvic girdle due to hormonal changes. It is estimated that more than two-thirds of pregnant women experience LBP,^[5]^ whereas ∼20% suffer from PGP.^[4]^ However, it can be difficult to clearly differentiate between LBP and PGP, and debate continues as to whether they should be considered together or separately.

In addition to pain, women with LBP or PGP commonly report disturbed sleep, difficulties attending normal daily activities, significant absenteeism from work, and residual symptoms postpartum.^[6,7]^ Some women also suffer considerable stress, with many worrying that their pain is a sign of problems with their developing baby.^[8]^ Despite this, women may receive little or no treatment to manage their condition.^[2]^ For those that do, common recommendations from medical professionals include education, pharmaceuticals, and exercises.^[4,5]^ Some women will also seek out complementary and alternative medicine (CAM) during pregnancy, which includes manual therapies such as massage, spinal manipulation, chiropractic, and osteopathy.^[9]^ Indeed research indicates that expectant women frequently use CAM therapies to manage their pregnancy-related conditions,^[10]^ and furthermore, midwives are often supportive.^[11]^

In the last 10 years, a number of authors have examined the evidence for a broad range of interventions to manage pregnancy-related LBP and PGP. A Cochrane review considered randomized controlled trials (RCTs) of any treatment (conventional or CAM), or combination of treatments.^[5]^ The reviewers found low-quality evidence that land-based exercise may be effective for back pain although no significant difference was found for pelvic pain. When back and pelvic pain were considered together there was moderate-quality evidence that a 12-week exercise program may be of benefit. The authors also reported that results from single clinical trials indicate that acupuncture or craniosacral therapy improves pregnancy-related pelvic pain, and osteomanipulative therapy or a multimodal intervention (manual therapy, exercise, and education) may also be helpful. Another evaluation considered combination of interventions (often with educational programs), exercise therapy, manual therapy, and material support.^[3]^ The reviewers concluded that exercise therapy and patient education had a positive effect on pain, but there was insufficient evidence regarding the effectiveness of manual therapies.

Several publications report on the effectiveness of a range of CAM interventions for pregnancy-related back pain. One paper considered a wide variety of therapies that included homeopathy, acupuncture, meditation, herbal medicine, and numerous manual therapies.^[12]^ The authors reported that acupuncture showed clinically important changes, and there were also some positive findings for osteopathy and chiropractic. However, the confidence in the results is rather low due to the methodological weakness of some of the studies. There are 2 reviews that focus specifically on the effectiveness of spinal manipulative therapy for pregnancy-related back pain.^[13,14]^ One included 5 randomized controlled trials, 1 cohort study, 2 case-controlled studies, and a small comparison study.^[13]^ The authors conclude that there is an emerging body of evidence to suggest spinal manipulative therapy may be an appropriate treatment option for some women, but high-quality clinical trials on safety and effectiveness are needed urgently. Results from the other review of 6 included studies that investigated chiropractic care found it was associated with improved outcomes.^[14]^ However, once again the quality of evidence was not sufficient to make any definitive statement as regarding the efficacy of spinal manipulation for pregnancy-related LBP.

The literature indicates that massage, chiropractic, and osteopathic treatments are commonly used by pregnant women to manage LBP and PGP.^[8,13]^ Although there are several reviews reporting on the interventions to manage pregnancy-related back and pelvic pain, interpreting the findings can be challenging due to the diverse mix of conventional and CAM therapies. The aim of this systematic review was to critically appraise and synthesize the best available evidence regarding the effectiveness of complementary manual therapies for the management of pregnancy-related LBP and PGP. The current review focuses specifically on a group of manual therapies commonly used by pregnant women: massage, chiropractic, and osteopathic treatments.

## 2 Methods

This systematic review is reported in accordance with the Preferred Reporting Items for Systematic reviews and Meta-Analyses (PRISMA) statement.^[15]^ A protocol was devised by the authors and used as a template for conducting the review according to the following:

### 2.1 Eligibility criteria

The interventions of interest were manual therapies including spinal mobilisation, spinal manipulation, massage, myofascial release, chiropractic, and osteopathy. The study population included pregnant women of any age and at any time during the antenatal period. The study designs that were considered for inclusion in the review were randomized controlled trials (RCT) and cluster randomized controlled trials. Possible comparators included usual care, no intervention or any other intervention including exercise, physiotherapy, or sham treatments.

### 2.2 Primary outcome

The primary outcome was LBP or PGP intensity. Secondary outcomes included pain-related disability, quality of life, medication, acceptance, and safety of women and children.

### 2.3 Information sources

The following electronic databases were searched from their inception until April 21st, 2015: PubMed/MEDLINE, Cumulative Index to Nursing and Allied Health Literature (CINAHL), Cochrane Library (CENTRAL), Allied and Complementary Medicine Database (AMED), PEDRO, PROQUEST, and Scopus. In addition, the reference lists of all identified records and articles were searched for further studies. The search was limited to full-text studies, published in the English or German language.

### 2.4 Search terms

The search strategy for PubMed can be found below. Database-specific search terms were developed, based on subject headings for the terms pregnancy, back pain, pelvic pain. The following search was constructed for PubMed: (Pregnancy[Mesh Terms] OR Pregnancy[Title/Abstract] OR Pregnant[Title/Abstract] OR Prenatal[Title/Abstract] OR Perinatal[Title/Abstract]) **AND** (“Back pain”[Mesh Terms] OR Sciatica[Mesh Terms] OR “Back Pain”[Title/Abstract] OR Sciatica[Title/Abstract] OR Lumbago[Title/Abstract] OR Radiculopathy[Title/Abstract] OR “Back Ache”[Title/Abstract] OR “Lumbar Pain”[Title/Abstract] OR “Pelvic Pain”[Mesh Terms] OR “Pelvic Pain”[Title/Abstract] OR “Pelvic Girdle Pain”[Title/Abstract] OR ((Sacral[Title/Abstract] OR Sacroili∗[Title/Abstract] OR Pelvis[Title/Abstract]) AND Pain[Title/Abstract]) **AND** (“Musculoskeletal Manipulations”[Mesh Terms] OR Manipulation∗[Title/Abstract] OR “Manual Therap∗”[Title/Abstract] OR “Manipulative Therap∗”[Title/Abstract] OR Chiropractic∗[Title/Abstract] OR Osteopath∗[Title/Abstract] OR Massage[Title/Abstract]) **AND** (“Randomized Controlled Trial”[Publication Type] OR “controlled clinical trial”[Publication Type] OR randomized[Title/Abstract] OR randomised[Title/Abstract] OR random∗[Title/Abstract] OR trial[Title/Abstract] OR group∗[Title/Abstract]) **NOT** (Animals[MeSH Terms] NOT humans[MeSH Terms]).

### 2.5 Study selection

Studies retrieved from the searches were screened for inclusion by 2 independent reviewers (HH, RL) using a template developed for the purposes of this review. Initially, titles and abstracts were screened according to the inclusion criteria. Following this initial screening, the full texts of records that appeared to meet the inclusion criteria were obtained and independently assessed for eligibility by the same reviewers. If there was doubt regarding the suitability of the study, the full text was assessed as well. A third reviewer (JA) was available to settle any disagreement between the reviewers. Where clarity or further information was required, attempts were made to contact the authors of the primary studies.

### 2.6 Data extraction

Two reviewers (RL, TS) independently extracted data using an extraction form specifically designed for the review, with any disagreements being resolved by a third reviewer (HC). The data extracted included details about the interventions, populations, study methods, and significant outcomes.

### 2.7 Risk of bias in individual studies

Two reviewers (RL, TS) independently assessed risk of bias using the standardized Cochrane Risk of Bias tool.^[16]^ Any disagreement was resolved by a third reviewer (HC). Where reported information was unclear or contradictory, or where important data was missing, attempts were made to contact the study author(s).

### 2.8 Assessment of overall effect size

Separate meta-analyses were conducted for comparisons of manual therapies to different control interventions using Review Manager 5 software (Version 5.3, The Nordic Cochrane Centre, Copenhagen). Random effects models were chosen if at least 2 studies assessing this specific outcome were available. For continuous outcomes, standardized mean differences (SMD) with 95% confidence intervals (CI) were calculated as the difference in means between groups divided by the pooled standard deviation using Hedges's correction for small study samples.^[16]^ Where no standard deviations were available, they were calculated from standard errors, confidence intervals, or *t*-values, or attempts were made to obtain the missing data from the study authors. Cohen's categories were used to evaluate the magnitude of the overall effect size: small, SMD = 0.2–0.5; medium, SMD = 0.5–0.8; and large, SMD>0.8.^[17]^

Dichotomous outcomes were analyzed by odds ratios (OR) and their respective CI. Odds ratios (OR) for safety of the interventions were calculated by dividing the odds of an adverse event in the intervention group (i.e., the number of participants with the respective type of adverse event divided by the number of participants without the respective type of adverse event) by the odds of an adverse event in the control group.

If statistical pooling was not possible due to heterogeneity, the findings were presented in narrative form, including tables and figures to aid in data presentation where appropriate.

### 2.9 Assessment of heterogeneity

Statistical heterogeneity between studies was analyzed using the *I*^2^ statistic, a measure of how much variance between studies can be attributed to differences between studies rather than chance. The magnitude of heterogeneity was categorized as low (*I*^2^ = 0–24%); moderate (*I*^2^ = 25–49%); substantial (*I*^2^ = 50–74%); or considerable (*I*^2^ = 75–100%).^[16,18]^ The chi-square test was used to assess whether differences in results were compatible with chance alone. Given the low power of this test when only few studies or studies with low sample size are included in a meta-analysis, a *P*-value ≤ 0.10 was used to indicate significant heterogeneity.^[17]^

### 2.10 Subgroup analyses

Subgroup analyses were planned for the primary outcome regarding: (1) the type of manual therapy intervention; and (2) duration and frequency of the intervention. Further subgroup analyses were conducted for trials that predominantly included women with back pain versus those who did not but measured back pain and disability.

### 2.11 Sensitivity analyses

To test the robustness of significant results, sensitivity analyses were conducted for studies with high versus low risk of bias at the following domains: selection bias (random sequence generation and allocation concealment), detection bias (blinding of outcome assessment), and attrition bias (incomplete outcome data). If present in the respective meta-analysis, subgroup and sensitivity analyses were also used to explore possible reasons for statistical heterogeneity.

### 2.12 Risk of publication bias

Risk of publication bias was assessed by 2 reviewers (RL, TS) for each meta-analysis that included at least 10 studies.^[19]^ Funnel plots—scatter plots of the intervention effect estimates from individual studies against the studies’ standard error—were generated using Review Manager 5 software. Publication bias was assessed by visual analysis, with roughly symmetrical funnel plots regarded as indicating low risk and asymmetrical funnel plots regarded as indicating high risk of publication bias.

## 3 Results

### 3.1 Study selection

**Figure 1:**
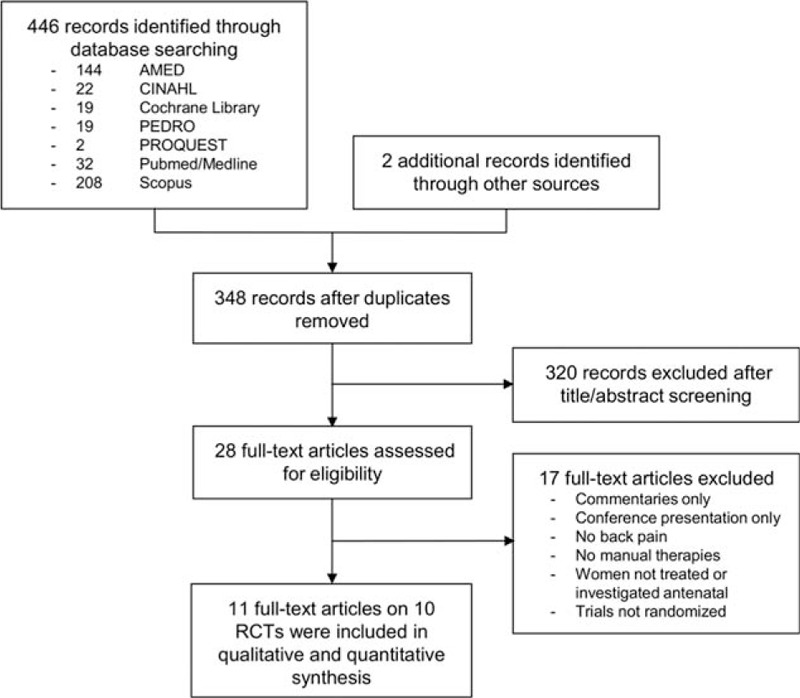
Study selection. Flow diagram summarizing the search strategy for this review.

Figure 1 shows the flowchart of study selection. The database searches retrieved 348 nonduplicate records, of which 320 were excluded after title/abstract screening. The remaining 28 full texts were assessed for eligibility, of which 17 articles were excluded because they were commentaries,^[20–24]^ conference presentations,^[25]^ did not investigate LBP or PGP during pregnancy,^[26–28]^ did not include manual therapies,^[29,30]^ women were not investigated or treated antenatal,^[31–33]^ or the trials were not randomized.^[34–36]^ The remaining 11 full-text articles, reporting on 10 studies, were included.^[37–47]^

### 3.2 Study characteristics

Study characteristics are presented in Table 1 . Of the 10 studies that were included 7 originated from the USA,^[39–46]^ and 1 each from Poland,^[37]^ Germany^[47]^ and Sweden.^[38]^ Pregnant women were recruited from antenatal classes,^[37]^ hospitals and obstetric clinics,^[38–46]^ and midwifes or gynecologists.^[47]^ The mean age of women in the studies ranged from 24 to 31 years with a median of 29 years. The average gestational age ranged from 21.0 to 30.0 weeks at baseline, with a median of 25.7 weeks. Only 2 studies reported specific inclusion criteria in terms of pain intensity (30 mm and 40 mm^[38]^ VAS), and only 1 specified that back pain has started during pregnancy.^[40]^

**Table 1:**
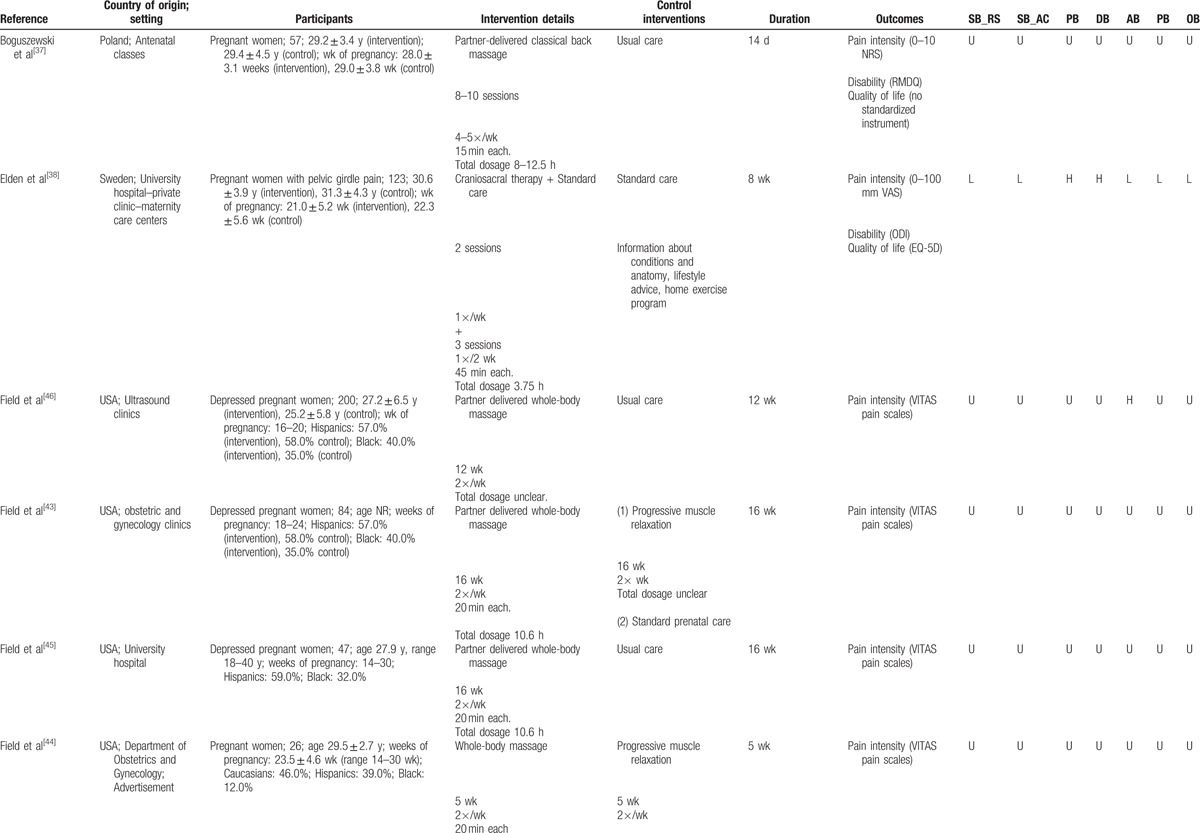
Characteristics of the included studies and risk of bias assessment using the Cochrane Risk of Bias tool.

**Table 1 (Continued):**
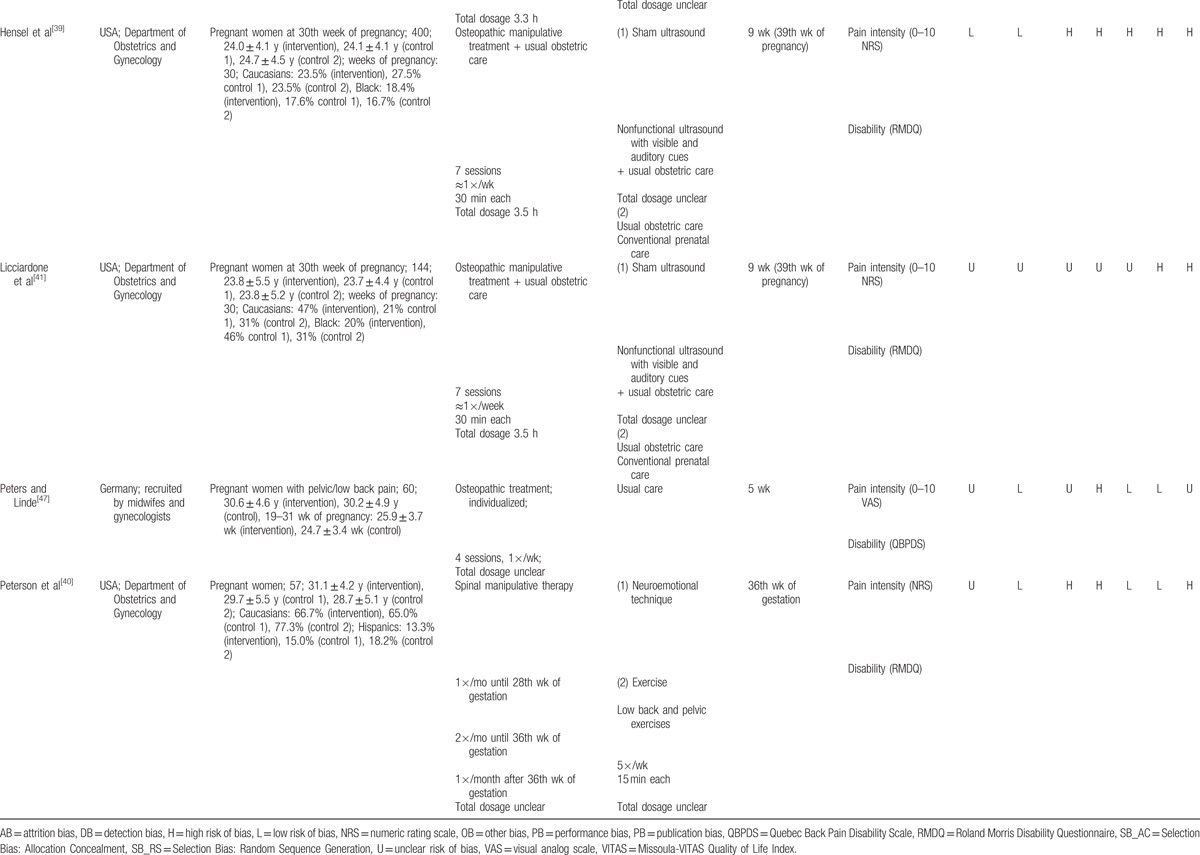
Characteristics of the included studies and risk of bias assessment using the Cochrane Risk of Bias tool.

Manual therapy interventions included craniosacral therapy,^[38]^ osteopathic manipulative treatment,^[39,41,42,47]^ chiropractic,^[40]^ massage,^[44]^ and partner-delivered massage.^[37,43,45,46]^ Duration of the interventions ranged from 2 to 16 weeks (median 7 weeks). Intervention parameters included 4 to 32 sessions (median 10 sessions), of 15 to 45 minutes duration (median 25 minutes), held at frequencies ranging from once per month to 5 times per week (median 1/week). Where possible to determine, dosage of interventions delivered ranged from 3.3 to 12.5 hours of massage therapy, 3.75 hours of chiropractic, and 3.5 hours of osteopathic manipulative treatment. Control interventions included usual care or standard prenatal care,^[37–39,41–43,45–47]^ progressive muscle relaxation,^[43,44]^ sham ultrasound,^[39,41,42]^ and exercise and chiropractic neuro-emotional techniques.^[40]^

A variety of outcome measures were used and the majority were self-reported. Pain intensity was measured using numeric rating scales,^[37,39–42]^ visual analogue scales,^[37,47]^ or the VITAS pain scale,^[43–46]^ which consists of a visual analog scale anchored with smiley/frowneys. Neck disability was measured by the Roland Morris Disability Questionnaire (RMDQ),^[37,39–42]^ the Oswestry Disability Index (ODI),^[38]^ and the Quebec Back Pain Disability Scale (QBPDS).^[47]^ Quality of life was measured using a validated score in 1 study only, using the EQ-5D.^[38]^

### 3.3 Risk of bias within studies

In general, selection bias of the included studies was unclear, with the exception of 2 studies reporting low bias of random sequence generation and 4 studies reporting low bias of allocation concealment. No study was assessed as low risk for performance or detection bias, and only 3 studies each had low attrition and reporting bias (Table 1 ).

### 3.4 Analysis of overall effects

**Figure 2:**
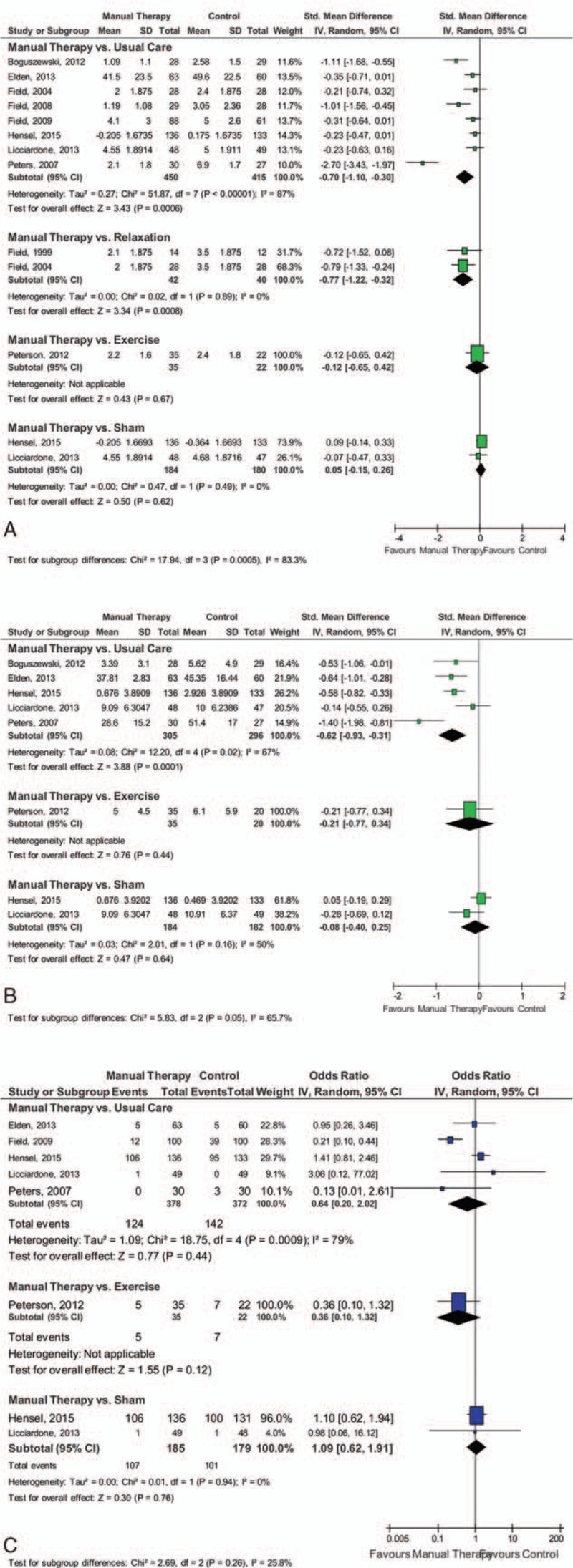
Forest plots. Manual therapies had positive effects on pain intensity when compared to both usual care and relaxation, and on pain disability when compared to usual care. However, there was no evidence for manual therapies when compared to sham interventions.

Meta-analyses revealed evidence for positive effects of manual therapy on pain intensity when compared to usual care (SMD = –0.70; 95% CI: –1.10, –0.30; *P* < 0.001) and relaxation (SMD = –0.77; 95% CI: –1.22, –0.32; *P* < 0.001); but not when compared to sham interventions (SMD = 0.05; 95% CI: –0.15, 0.26; *P* = 0.62) (Fig. 2A). Similarly, evidence for positive effects of manual therapy on pain disability were found when compared to usual care (SMD = –0.62; 95% CI: –0.93, –0.31; *P* < 0.001); but not when compared to sham interventions (SMD = –0.08; 95% CI: –0.40, 0.25; *P* = 0.64) (Fig. 2B). No meta-analysis could be conducted for quality of life due to the paucity of data. Acceptability (i.e., number of dropouts), did not differ between manual therapy and usual care (OR = 0.64; 95% CI: 0.20, 2.02; *P* = 0.44) or sham interventions (OR = 1.09; 95% CI: 0.62, 1.91; *P* = 0.76) (Fig. 2C).

### 3.5 Subgroup analysis

Due to the paucity of eligible trials no subgroup analysis regarding the type of manual therapy intervention, and the duration and frequency of the intervention could be conducted.

Excluding those trials which primary target population was not back pain^[43,45,46]^ the effect on pain intensity remained significant for manual therapy compared to usual care (SMD = –0.85; 95% CI: –1.47, –0.23; *P* = 0.007). Only 1 study was left for the comparison to relaxation,^[44]^ without a significant effect. No changes were found for disability, or acceptability between manual therapy and usual care (OR = 1.27; 95% CI: 0.78, 2.09; *P* = 0.34).

### 3.6 Sensitivity analysis

The effects of manual therapy compared to usual care on pain intensity and pain disability as well as acceptability of the intervention did not change substantially when only RCTs with low risk of selection bias were considered, but heterogeneity was reduced (Table 2). Sensitivity analyses for manual therapy compared to relaxation or sham intervention could not be computed due to lack of data. As no study had low risk of detection bias, no sensitivity analysis for this type of bias could be computed.

**Table 2:**
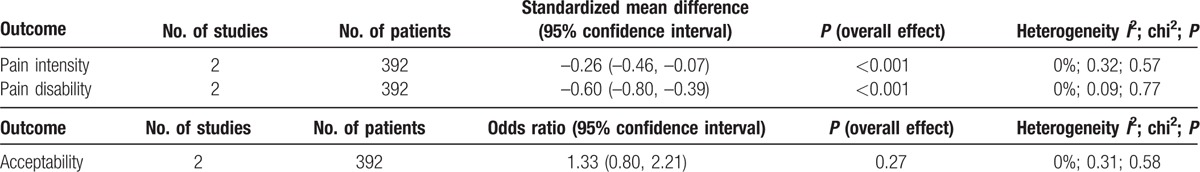
Sensitivity analysis: effects of manual therapy versus usual care in studies with low risk of selection bias.

### 3.7 Risk of bias across studies

**Figure 3:**
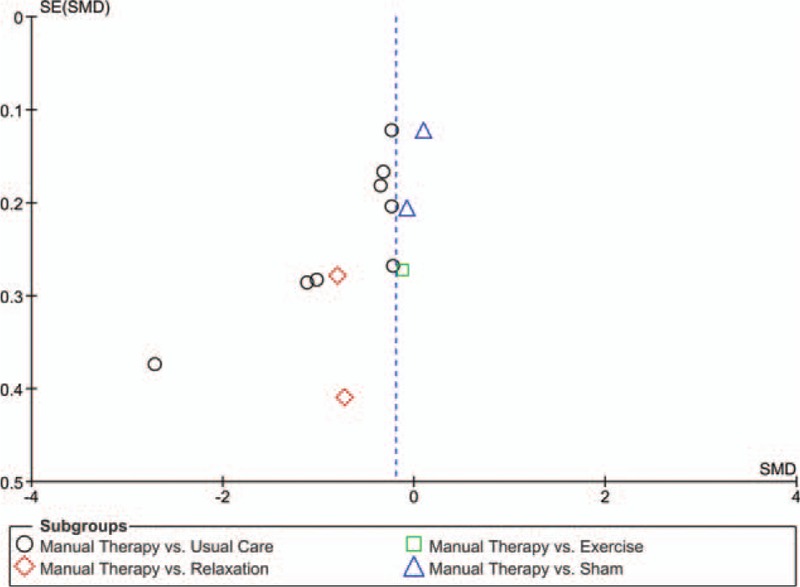
Funnel plot. The funnel plot for pain intensity indicated a possible risk of publication bias.

The funnel plot for pain intensity was asymmetrical, indicating a possible risk of publication bias (Fig. 3). Since <10 studies were included in the remaining meta-analyses, no further funnel plots were computed.

### 3.8 Safety

Only 3 studies reported safety, but no meta-analysis could be conducted. Although 1 study reported no health problems during the massages,^[37]^ a case of early contractions in the control group was reported in another study.^[47]^ A third study stated that the massage group had fewer obstetric complications, their newborns had fewer postnatal complications, less premature births and infants required less ventilatory assistance.^[44]^

## 4 Discussion

The aim of this review was to determine the effectiveness of manual therapies for pregnancy-related LBP and PGP. Ten studies were included in the meta-analysis. The specific therapies included craniosacral therapy, chiropractic neuro-emotional techniques (NET), osteopathic manipulative treatment, and massage. Craniosacral therapy involves gentle manipulations of the skull and NET is a mind-body approach used by some chiropractors as a stress-reduction technique. Osteopathic treatments may include the manipulation of joints and the application of pressure on the skin through a “thrust” technique, whereas massage involves the manipulation of soft tissue only.

The results indicated a moderate treatment effect of manual therapies for decreasing pain intensity compared to usual care and relaxation, and a moderate effect on pain disability compared to usual care. However, no positive effects for manual therapies were found for either pain intensity or pain disability when compared to sham interventions. Specifically, positive effects were found for pain intensity for osteopathy^[47]^ and partner delivered massage compared to usual care with an overall positive effect when the therapies were combined. Partner delivered massage^[48]^ had a positive effect on pain intensity experienced by depressed pregnant women when compared to relaxation, and there was an overall positive effect when the therapies were combined. This review also found positive effects for pain disability for craniosacral technique and osteopathy^[47]^ compared to usual care, and an overall positive effect when the therapies were combined. Acceptability did not differ between manual therapies and usual care or sham interventions. These findings are consistent with a recent Cochrane review suggesting moderate-quality evidence from individual studies that indicate osteopathic manipulative therapy significantly reduced LBP and disability whereas craniosacral therapy improved pregnancy-related lumbo-pelvic pain more than usual care.^[5]^

The limited effectiveness of manual therapies over passive, self-delivered treatments, but not over the active, therapist-delivered treatments suggest the possibility of a therapist effect. As such, the process of having a therapy delivered to a person by a therapist or a significant other person such as the partner may influence the perceived effectiveness of the treatment. Researchers in the United Kingdom evaluated the size and influence of the “practitioner effect” in 3 randomized trials for patients with lower back or neck pain.^[49]^ Findings from this study indicate that a “practitioner effect” does indeed exist which highlights not only the importance of the expertise of the individual therapist but the significance of the therapeutic exchange itself. Some of the therapies evaluated in this review were delivered by the woman's partner^[49–51,50]^ and this relationship may have a significant influence over the effectiveness of the therapy being delivered. Furthermore, active control treatments were limited to sham ultrasound.^[39,41]^ Investigation into the delivery of both authentic and sham versions of a manual therapy treatment to this population would allow further investigation into the extent of the therapy versus the therapist effect on treatment outcomes. It is important that the role of the “therapist” and the impact it has on effectiveness of an intervention is considered when designing and evaluating pain studies.

Additionally, treatment dosage of the manual therapies may have influenced results. Significantly effective intervention doses of massage ranged from 8 to 12.5 hours compared to usual care,^[37,45]^ and 10.6 hours compared to matched doses of progressive muscle relaxation.^[43]^ In comparison, lower doses of 3.5 hours of osteopathic manipulative treatment had no effect on pain intensity or disability compared to a matched dose of sham ultrasound.^[39,41]^ As previous findings suggest that osteopathic manipulative treatment significantly reduces LBP in nonpregnant participants,^[51]^ more research into the potential confounding effects of dosage and therapist attention is required.

The findings of this review have important implications for maternity care practitioners and the women they care for who are suffering with LBP and PGP. Despite some concerns regarding the use of manual therapies by pregnant women, Oswald et al^[8]^ assert that very few adverse effects have been reported in the literature. Findings from the few studies included in the current review^[37,44,47]^ that reported safety are consistent and suggest complementary manual therapies as a safe and effective option during pregnancy compared to no treatment at all. However, the effectiveness of these therapies in comparison to other physical or therapist-administered interventions was not possible to determine due to the lack of high-quality research with active control groups. Therefore, in line with previous research,^[3]^ the review did not find sufficient evidence to recommend the use of complementary manual therapies for pregnancy-related LBP and PGP.

This review has identified several areas in which quality of evidence may be improved. Most studies provided insufficient information to determine overall risk of bias, resulting in most domains of bias being rated as unclear. Areas of evident high risk of bias related to blinding of participants, and therefore to blinding of outcome assessment due to the use of self-reported outcomes measures. Differentiation in bias between studies was most evident in the domains of incomplete outcomes data and selective reporting, and least evident for selection bias.

Areas of focus for future research include a more robust, active comparator such as exercise or physical therapy as the control intervention, and investigation into the role of the therapist in the treatment effect. A uniform level of treatment dosage for experimental and control interventions (frequency, duration) within manual therapy studies as well as controlled dose variations may assist in identifying dosage effects of treatments. Additionally, the identification of clinically effective therapy doses will allow an economic assessment of the cost-effectiveness of various manual therapy interventions, providing evidence for their cost-effective integration into current hospital-provided pregnancy care.

A limitation to the findings of the current review is the inclusion of trials which investigate back pain outcomes in pregnant women,^[43,45,46]^ even though their primary aim is to examine the efficacy of manual therapy for depression. However, effects remained significant even after exclusion of those studies in subgroup analyses. A further limitation is the diversity of treatment types and dosage of the manual therapies included in the meta-analysis. Although this has been addressed to an extent by the subanalysis of active versus passive control groups, it has not been possible to control for dosage, especially as not all studies reported session duration of treatments. Another limitation is the type of sham control used in some of the included studies. The gold standard for RCTs requires double blinding of both investigator and participants and the use of an appropriate placebo. Although it is not possible to blind the persons who deliver the interventions in a manual therapy trial, blinding of evaluators is possible, which together with more active sham and control treatments will contribute to improved trial methodology. Blinding of patients on the other hand might be difficult if not impossible; however, the use of sham to blind patients receiving osteopathy^[51,52]^ or craniosacral therapy^[53]^ has been successful in previous trials, and they might be suitable for certain types of manual therapies. The sham controls used in studies in the current review, however, were not sham controls of the active intervention but of a sham of ultrasound,^[39,41]^ thus limiting the use of sham as a form of participant blinding. For Swedish massage, no sham might be possible at all; however, the potential bias might be reduced or even eliminated by designing a control intervention of equal value with comparable attention and care. The paucity of data for all outcomes is limiting the validity of this meta-analysis, as is the potential bias introduced by language restrictions which might have led to inclusion of studies predominantly from the West.

## 5 Conclusion

There is currently limited evidence to support the use of manual therapies including massage and osteopathic manipulative treatment as an option for managing LBP and PGP during pregnancy. Current research is associated with a risk of publication and methodological biases, and lack of robust control comparisons. Further high-quality research is needed to determine causal effects, the influence of the therapist on the perceived effectiveness of treatments, and adequate dose–response of manual therapies on LBP and PGP outcomes during pregnancy.
